# Bacterial taxonomic and functional changes following oral lyophilized donor fecal microbiota transplantation in patients with ulcerative colitis

**DOI:** 10.1128/msystems.00991-25

**Published:** 2025-09-15

**Authors:** Shreeya S. Raich, Marwan E. Majzoub, Craig Haifer, Sudarshan Paramsothy, Md Mushahidul Islam Shamim, Thomas J. Borody, Rupert W. Leong, Nadeem O. Kaakoush

**Affiliations:** 1 School of Biomedical Sciences, Faculty of Medicine and Health, UNSW Sydney 7800, Sydney, New South Wales, Australia; 2 School of Clinical Medicine, Faculty of Medicine and Health, UNSW Sydney 7800, Sydney, New South Wales, Australia; 3 Department of Gastroenterology, St Vincent’s Hospital, Sydney, New South Wales, Australia; 4 Concord Clinical School, University of Sydney 4334, Sydney, New South Wales, Australia; 5 Department of Gastroenterology, Concord Repatriation General Hospital 2659, Sydney, New South Wales, Australia; 6 Centre for Digestive Diseases 94792, Sydney, New South Wales, Australia; University of Southampton, Southampton, United Kingdom

**Keywords:** fecal microbiota transplantation, microbiome, metagenomics, ulcerative colitis, pathway, resistome

## Abstract

Oral lyophilized fecal microbiota transplantation (FMT) can induce remission in patients with active ulcerative colitis (UC); however, our understanding of how this form of FMT alters the patient microbiome remains limited. Here, we analyzed data from a recent randomized, double-blind, placebo-controlled clinical trial of FMT in UC to assess donor species colonization and factors responsible for efficacy using this form of therapy. The gut microbiome of donors and patients was profiled longitudinally using deep shotgun metagenomic sequencing, and microbiome diversity, species-genome bin presence, functional profiles, and the resistome were studied. The gut microbiome of patients treated with oral lyophilized FMT significantly increased in species-genome bin richness and shifted in composition toward the donor profiles; this was not observed in patients receiving placebo. While species-genome bin richness was not associated with clinical response in this trial, we identified donor- and patient-specific features associated with the induction of remission and maintenance of response. However, the presence of a *Clostridium* species-genome bin, as well as L-citrulline biosynthesis contributed by *Alistipes* spp., was seen in responders treated by either donor. Several of the above outcomes were found to be consistent when data were analyzed at the level of metagenome-assembled genomes. FMT was also found to deplete the resistome within patients treated with antibiotics to levels lower than the UC baseline. Single donor oral lyophilized FMT substantially modifies taxonomic diversity and composition as well as microbiome function and the resistome in patients with UC, with several features identified as strongly linked to response regardless of the donor used.

**IMPORTANCE:**

There is a limited amount of work examining the effects of oral lyophilized fecal microbiota transplantation (FMT) on the microbiome of patients with ulcerative colitis (UC), and less so studies examining species-level dynamics and functional changes using this form of FMT. We performed deep shotgun metagenomic sequencing to provide an in-depth species-genome bin-level analysis of the microbiome of patients with UC receiving oral lyophilized FMT from a single donor. We identified key taxonomic and functional features that transferred into patients and were associated with clinical response. We also determined how FMT impacts the resistome of patients with UC. We believe these findings will be important in ongoing efforts to not only improve the efficacy of FMT in UC but also allow for the transition to defined microbial therapeutics, foregoing the need for FMT donors.

## INTRODUCTION

The human gut microbiome functions to support host digestion and absorption of foods and nutrients, immune system homeostasis, and mucosal layer integrity, among other roles (1). Disruptions to its diversity, composition, or function from an individual’s baseline healthy state, termed dysbiosis, can lead to immune system dysfunction and inflammation, and consequently, disease development (2). Dysbiosis is an attribute of the inflammatory bowel disease, ulcerative colitis (UC), which is believed to play an important role in its etiopathogenesis (3). Some characteristics of the microbiome changes in UC include a decrease in species diversity, increases in the relative abundances of pathobionts (opportunistic microbes that can become harmful under specific conditions), and decreases in short-chain fatty acid producers, all of which lead to functional variations away from homeostasis with the host (4–6).

Treatment of patients with UC, a remitting-relapsing disease, encompasses immunomodulators regardless of severity (7). Current immune therapies provide relief, but these can result in side effects, be ineffective, or lose efficacy over time (7). Microbiome manipulation has been proposed as an alternative strategy to treatment that does not require immune inhibition and its resultant side effects. One form of manipulation, antibiotic therapy, has a limited effect on disease (8) and is not particularly helpful in that it would drive more dysbiosis and antibiotic resistance in such a chronic disease. An alternative, fecal microbiota transplantation (FMT), which aims to restore microbiome balance through the transplant of a properly functioning microbial community, has been trialed more recently and shown modest efficacy (primary endpoint is achieved in approximately 30%–50% of patients) (9–12).

Along with other design factors, such as mode of delivery, formulation, and dosage, taxonomic and functional features of the donors and patients have been associated with FMT success or failure (13–16). Species richness of donors and patients post-treatment is a key property associated with response to FMT in UC (16, 17). While the taxonomic features are less consistent across trials and geography, they likely converge to lesser-explored functions depleted or absent in patients (18, 19). Despite these advances made in our understanding of FMT in UC, there are several aspects that remain underexplored. One that is highly relevant is the effect of oral, fridge-stable formulations of FMT at the bacterial species-genome bin (SGB), metagenome-assembled genome (MAG), and functional level. The importance of this relates to the future of microbial therapies, given the need to progress toward these forms of live microbial products for mass utility. However, the information on this is lacking because not many trials that have employed this form of FMT in UC have combined this with deeper shotgun sequencing that is required for this level of analysis. Another highly relevant aspect is the impact of FMT on the patient’s resistome, the pool of antibiotic resistance genes within a microbial community. FMT is increasingly being administered following antibiotic pre-treatment of patients, as this is reported to improve donor strain engraftment (20). Thus, it is crucial to understand the impacts of this combined therapy and if FMT can restore lower levels of antimicrobial resistance in the patient’s microbiota.

We recently conducted a double-blind, randomized, placebo-controlled clinical trial using oral lyophilized FMT to treat patients with active mild-to-moderate UC (21). Treatment included a triple regimen of antibiotics for 2 weeks prior to single-donor FMT for 8 weeks, and this proved successful, showing a significantly higher ability to induce remission in patients than placebo. Here, we analyzed previously generated deep shotgun metagenomic sequencing to profile the bacterial component of the gut microbiome of patients and donors using a reference-based analysis at the SGB level and an MAG-based analysis to understand the effects of this form of FMT on patients with UC.

## MATERIALS AND METHODS

### Study cohort

This trial was double-blind, randomized, placebo-controlled and was conducted at two Australian centers between 20 May 2019 and 24 March 2020. Inclusion criteria were patients aged 18–75 years with active ulcerative colitis defined as a total Mayo score of 4–10 and a Mayo endoscopic subscore ≥1. After 2 weeks of antibiotics (amoxicillin, metronidazole, and doxycycline), patients (*n* = 35) were randomly assigned to receive either oral lyophilized FMT (*n* = 15) or placebo (*n* = 20) capsules for 8 weeks. Each FMT capsule contained 0.35 g of lyophilized stool. Placebo capsules were double encapsulated to replicate the FMT capsules and contained an inert brown powder made of colloidal anhydrous silica EX133/0, magnesium stearate EX002/0, calcium hydrogen phosphate dihydrate EX003/0, maltodextrin EX010/0, and croscarmellose sodium. Dosing consisted of six capsules four times a day for 1 week, six capsules two times a day for 1 week, and then six capsules daily for 6 weeks. Eight of the 15 patients (53%) receiving FMT and three of the 20 patients receiving placebo achieved the primary outcome (odds ratio 5.0, 95% CI 1.8–4.1, *P* = 0.027), which was corticosteroid-free clinical remission with endoscopic remission or response (total Mayo score ≤ 2, all subscores ≤ 1, and ≥1 point reduction in endoscopic subscore) at week 8. At week 8, a subset of patients who responded to FMT (*n* = 10) were randomly assigned to either continue (*n* = 4) or withdraw (*n* = 6) FMT for a further 48 weeks. For the maintenance arm, FMT was administered open label and at a dose of two capsules daily. All four patients who received FMT were in clinical, endoscopic, and histologic remission at week 56 compared with none of the patients who had withdrawn from FMT. Samples in this study included patients at baseline (*n* = 31), patients following antibiotics (*n* = 32), patients at week 1 (*n* = 33), week 2 (*n* = 30), week 3 (*n* = 26), week 4 (*n* = 24), or week 8 (*n* = 23) treatment, patients at week 20 (*n* = 5), week 32 (*n* = 9), week 44 (*n* = 3), and week 56 (*n* = 5) treatment or withdrawal, donor 1 samples (*n* = 12), and donor 2 samples (*n* = 17).

All available patient and donor stool samples from the LOTUS clinical trial (*n* = 250) were profiled using shotgun metagenomic sequencing (21–23). As reported previously (23), DNA was obtained from fecal samples using the QIAamp PowerFecal DNA Kit (Qiagen; Chadstone, VIC, Australia). DNA sequencing was performed at the Ramaciotti Centre for Genomics (UNSW Sydney), where libraries were generated using Illumina DNA prep kits (Illumina; Melbourne, VIC, Australia) and sequenced on an Illumina NovaSeq 6000 using 2 × 150 bp chemistry (S4 run; DNA input of 500  ng). Library preparation and sequencing were performed for two negative controls that generated no data. The bacterial component of the gut microbiome in the donors and the phageome of patients and donors were assessed previously (22, 23).

### Raw sequencing data analysis

Raw sequencing reads belonging to the same sample were concatenated into a single set of forward and reverse fastq files and were then quality trimmed using the Read_qc module within MetaWRAP version 1.3.2 to trim adaptors and remove human contamination with default parameters. Quality filtered reads were analyzed using MetaPhlAn4 (database vOct22) to profile the microbial communities and generate tables with relative abundances of taxa classified to the SGB level using a marker-based analysis (species level in MetaPhlAn4 output) (24). HUMANn3 version 3.0.1 was employed for functional analysis (25), where count tables of MetaCyc pathways and gene families were generated for downstream analysis.

Antibiotic-resistant genes (ARGs) were quantified using the ARG annotation pipeline ARGs-OAP 3.2.2 (http://smile.hku.hk/SARGs/) (26, 27) through a two-stage process. During stage one, the stage_one.py script was executed to screen for ARG-like and 16S ribosomal RNA gene sequences. Stage two of the pipeline included aligning the ARG sequences predicted from stage one with the metadata against the SARG reference databases by executing the stage_two.py script. The abundances of types, subtypes, and genes for ARGs were reported for each metagenomic sample. ARG abundances from metagenomic data sets are quantified using the equation provided in Yin et al. (27). Abundances are normalized against an estimated cell number by mapping against a database containing essential single-copy marker genes.

### Diversity and statistical analysis

The alpha diversity measure, Margalef’s richness at the SGB level, was calculated using Primer-e version 6. SGB relative abundances were center-log transformed using the Tjazi package (clr_lite option with method set at logunif and 1,000 replicates) in R version 4.3.2. Presence-absence or square-root transformations, creation of resemblance matrices of Aitchison distances (Euclidean distance on clr transformed data) or Bray-Curtis dissimilarities, principal coordinate analysis (PCO), permutational multivariate ANOVA (PERMANOVA), and permutational analysis of dispersions (PERMDISP) were performed using Primer-e version 6. The engraftment analysis was carried out using R version 4.3.2. The heatmaps were visualized using GraphPad Prism version 10, and the Venn diagrams were visualized using R packages “Venn.diagram,” “grid,” and “ggplot2.” All other statistical analyses as described, including testing for normal distribution using the Shapiro-Wilk test, were performed using GraphPad Prism version 10.

To assess donor SGB presence in patients, donor-only and patient-only SGBs were defined through a presence/absence analysis between patients and their respective donors, where presence was defined as a relative abundance >0%. SGBs found in both patients and donors were classified as mixed and excluded. Next, the presence of donor-only SGBs in patients following FMT was determined. The presence of patient-only SGBs was also assessed relative to the time points (following antibiotics, induction FMT, and maintenance FMT).

### Generation, quality checking, and classification of metagenome-assembled genomes

Raw sequencing reads belonging to the same sample were concatenated into a single set of forward and reverse fastq files and were then quality trimmed using the Read_qc module within MetaWRAP version 1.3.2 (28) to trim adaptors and remove non-microbial contamination. Reads for each sample were then assembled independently using the assembly module in MetaWRAP version 1.3.2 (SPAdes v3.1, --meta option) (29) with default parameters. MAGs were generated for each sample with default parameters from the assemblies using MetaBAT version 2 (30), MaxBin version 2 (31), and CONCOCT (32) and subsequently refined using MetaWRAP version 1.3.2. To produce a count table across all samples, MAGs were de-replicated at two thresholds, >99.99% and >97% average nucleotide identity (ANI). The completeness and contamination of MAGs were determined with CheckM (33), and MAGs were classified using PhyloPhlAn3 version 3.0.60 (34). Two types of analysis were performed, one considering all MAGs, and another including only high and moderate quality MAGs (>50% complete and <10% contamination). Genes from the MAGs were predicted using Prodigal version 2.6.3 (35), and functional annotations were performed using eggNOG-mapper version 2.1.12 (36). Gene functions were predicted by mapping query sequences against the eggNOG version 5.0 database (37), using precomputed clusters and phylogenies. Functional enrichment analysis was performed with the enrichMKEGG function within the clusterProfiler package (version 4.16.0) using R version 4.5.1.

## RESULTS

### Single donor oral lyophilized FMT modifies the microbiome of patients with UC at the species-genome bin level

**Fig 1:**
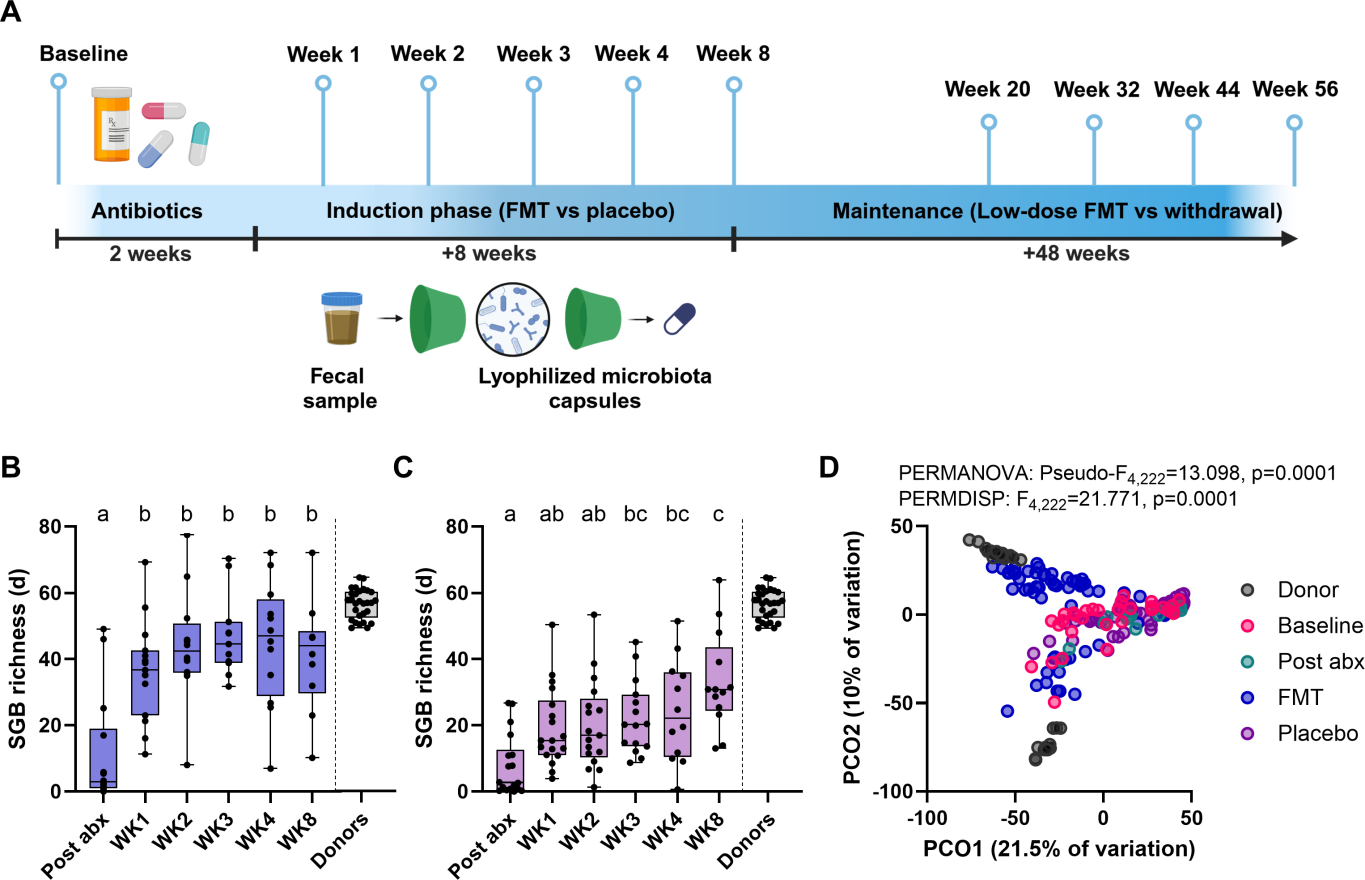
Oral lyophilized fecal microbiota transplantation alters the microbiome of patients with ulcerative colitis. (**A**) Trial design and sampling. Changes in species-genome bin richness following (**B**) FMT or (**C**) placebo in patients with ulcerative colitis treated with antibiotics. Differences were tested using two-way ANOVA with a Tukey’s multiple comparisons test, combining data from both treatments (FMT and placebo). Only within-group statistics are presented as a compact letter display (different letters, significantly different; same letters, not significantly different), with the remaining statistics included in Table S3. Donors were excluded from statistical tests as they represent longitudinal samples from two individuals. (**D**) Changes in patient SGB composition following antibiotics, donor FMT, and placebo. Ordination plot is a principal coordinate analysis on Aitchison distances, with inter-group differences tested using PERMANOVA and PERMDISP. Pairwise comparisons are included in Table S4. Post-abx, post-antibiotics; WK, week; PCO1, principal coordinate axis 1; and PCO2, principal coordinate axis 2.

We analyzed shotgun metagenomic data from the LOTUS clinical trial that treated patients with UC with single-donor oral lyophilized FMT (*n* = 15) or placebo (*n* = 20) to study taxa dynamics and microbial functional changes following this form of therapy. Our first taxonomic analysis was based on the presence/absence of SGBs as determined through their relative abundances (Table S2). These were estimated with MetaPhlAn4 through the detection and estimation of coverage of species-specific marker genes established from a large collection of SGBs (24). A complementary assembly-based analysis is presented later. As the patients received antibiotic treatment prior to FMT, we also examined the impact this combination therapy had on the resistomes. The data were derived from fecal samples collected from patients at baseline, after antibiotics, as well as during induction treatment with FMT (weeks 1, 2, 3, 4, and 8) (Fig. 1A). Furthermore, it included samples collected from a treatment maintenance phase where a subset of patients in remission received low-dose FMT or were withdrawn from therapy (weeks 20, 32, 44, and 56) (Fig. 1A). The data set also included longitudinal microbiome profiling of the two individual donors who contributed the FMT to treat patients (donor 1: *n* = 4 patients and donor 2: *n* = 11 patients). The average read depth of the metagenomic data following quality filtering and removal of host contamination was 36,799,073 reads (standard error of mean: 1,186,931 reads).

We found that oral lyophilized FMT led to an immediate significant increase in SGB richness post-antibiotics, which was maintained until the conclusion of induction therapy at week 8 (Fig. 1B). In contrast, while the SGB richness of patients who received placebo did recover following antibiotics, this process took longer (weeks 3–4), and even at the conclusion of therapy at week 8, it remained, on average, lower than that of patients on FMT (FMT: 41.06 vs placebo: 33.22) (Fig. 1B and C; Fig. S1A and B; Table S3). Moreover, we observed a clear shift in bacterial SGB composition (beta diversity based on Aitchison distances) in patients treated with FMT, but not in patients on placebo, and this shift was toward the respective donor that the patients received (Fig. 1D; Table S4). Sample clustering suggested that the microbial community of one of the donors (donor 2) had an improved ability to engraft into patients, given that patient samples post-FMT clustered closer to that donor’s samples (Fig. 1D). This was confirmed by testing the distances between donor samples and patient sample post-FMT, with patients receiving donor 2 having a significantly lower distance from the donor samples (Fig. S1C). Overall, this confirmed that oral lyophilized FMT capsules derived from single donors have a robust capacity to influence the microbiome of patients with UC following antibiotics.

### Signatures associated with response to FMT are donor- and patient-specific

**Fig 2:**
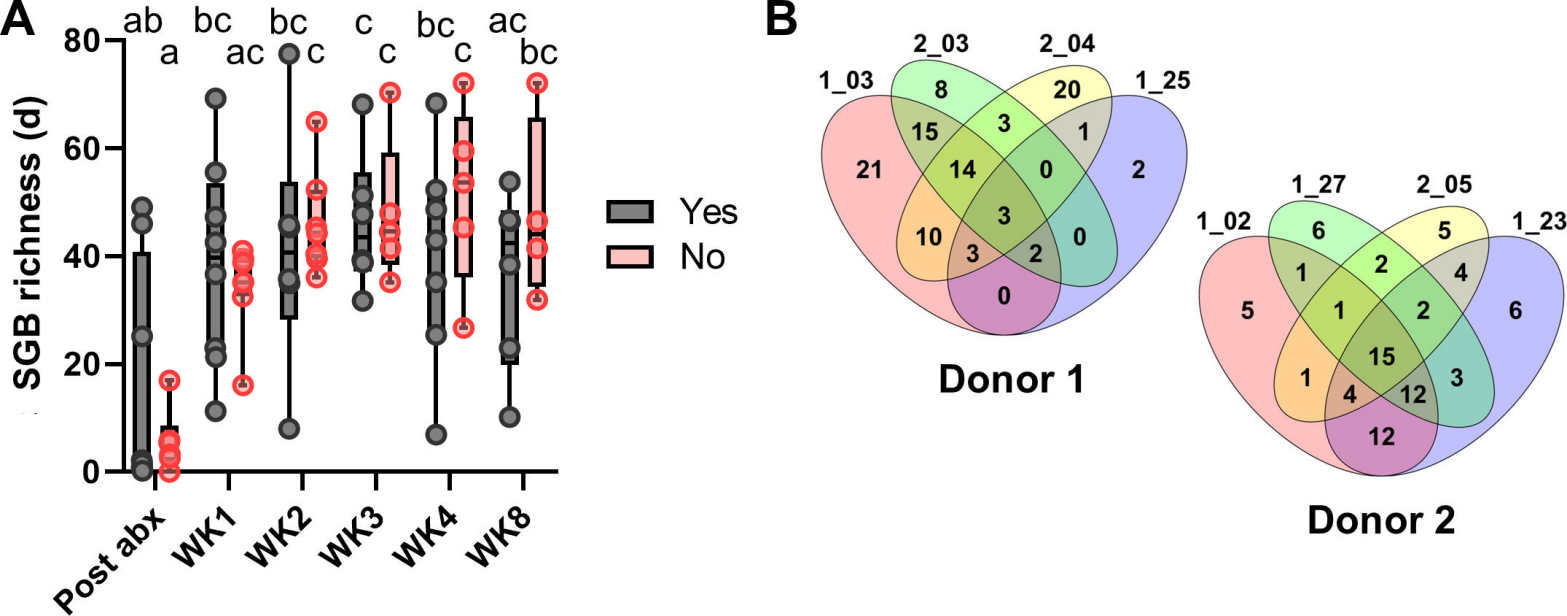
Taxonomic features associated with clinical response in patients with ulcerative colitis receiving oral lyophilized FMT. (**A**) Changes in species genome-bin richness in responders (Yes) and non-responders (No) to FMT in patients with UC treated with antibiotics. Differences were tested using two-way ANOVA with a Tukey’s multiple comparisons test. Statistics are presented as a compact letter display (different letters, significantly different; same letters, not significantly different). (**B**) Venn diagrams of donor-only SGBs that were detected in responders post-FMT. Donor-only SGBs were defined as those with no evidence of detection in any of the four responders receiving that donor. Numbers above the ovals refer to de-identified patient codes. Post-abx, post-antibiotics; WK, week; Yes, responder; and No, non-responder.

**Fig 3:**
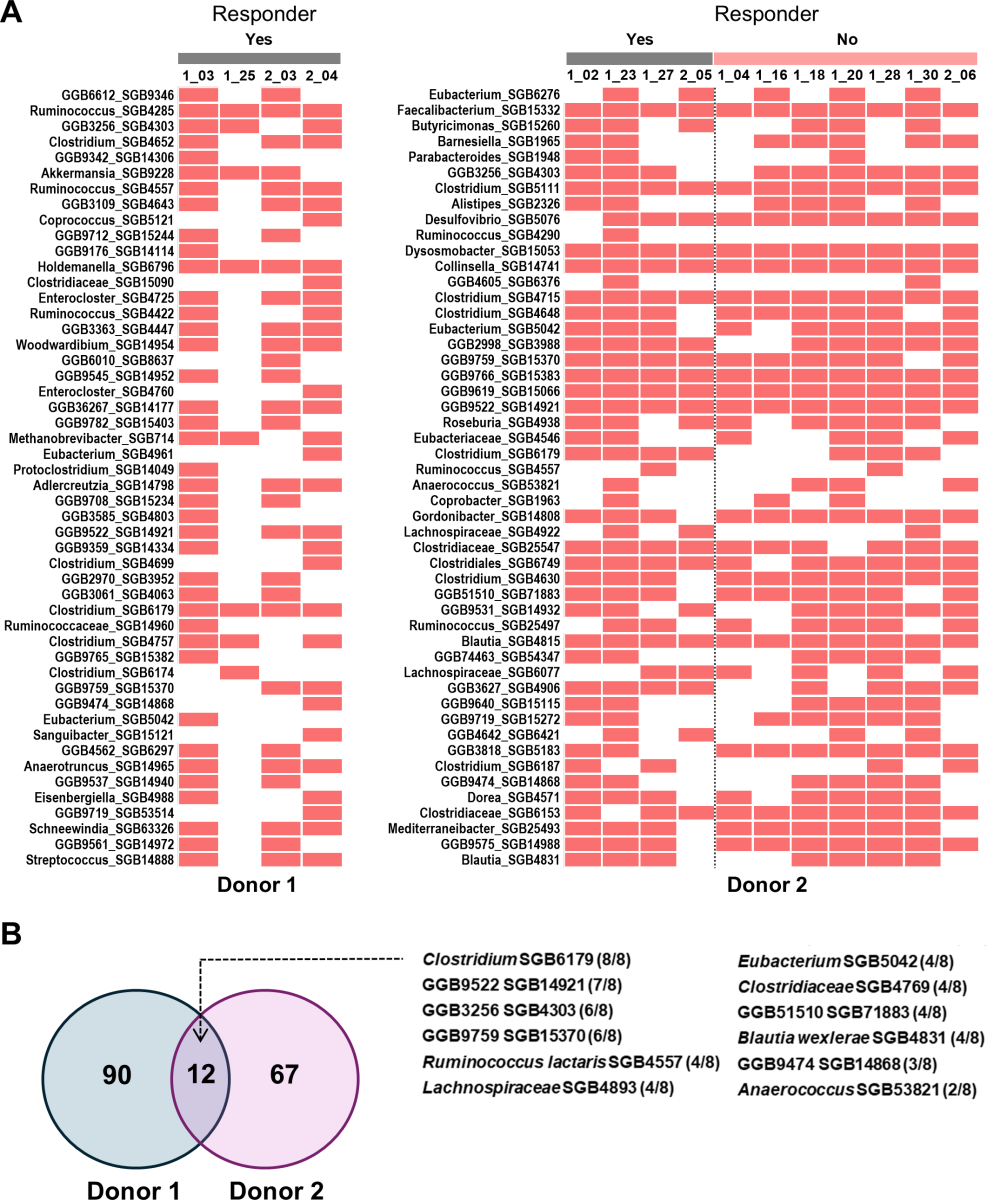
Individual taxa associated with clinical response in patients with ulcerative colitis receiving oral lyophilized FMT. (**A**) Heatmap of detection of top 50 donor-only SGBs in patients following FMT as ranked by mean relative abundance. Red, present. The full taxonomy of the SGBs is made available in Table S5. (**B**) Venn diagram of donor-only SGBs with evidence of transfer into responsive patients. The 12 SGBs listed were common to both donors, with the number of responders they were detected in included in parentheses. Numbers above the columns refer to de-identified patient codes. Yes, responder; No, non-responder.

We then assessed if any taxonomic properties could distinguish between responders (*n* = 8 total, four from each donor) and non-responders (*n* = 7, all from one donor) to FMT. In this trial, neither SGB richness (Fig. 2A) nor beta diversity at the SGB classification (all *P* > 0.11) was different between the two patient groups at any week of treatment. However, the latter would likely have been affected by the donor-specific impact on the patients (Fig. 1D) since each donor contributed four responders and donor 1 did not contribute non-responders. To account for this, we stratified patients by donor received and explored SGB-level features that may be associated with response. To perform this analysis, we assigned SGBs as donor-only, patient-only, or both based on their presence or lack thereof in each donor, as well as all responder patients for that donor at baseline and post-abx. Here, donor-only SGBs represented those that were present in donor 1 or 2 and not present in any of the patients they treated at baseline and post-abx. Patient-only SGBs were detected in patients and not present in the treating donor at any of the sampled time points. We then determined if donor-only SGBs were present in responders following FMT (Fig. 2B). The overlap among donor-only SGBs defined for each of the donors (donor 1 and donor 2) that were subsequently detected in each of the four responders post-FMT was plotted (Fig. 2B). We identified three donor-only bacterial SGBs *Holdemanella porci* SGB6796*, Ruminococcus bromii* SGB4285, and *Clostridium* SGB6179 that were detected in all four patients receiving donor 1 FMT (Fig. 2B and 3A). We also found 19 donor-only SGBs, including *Akkermansia muciniphila* SGB9228, *Methanobrevibacter smithii* SGB714, *Clostridium* sp. AM49 4BH SGB4757*, Anaerotruncus massiliensis* SGB14965, and *Ruminococcus lactaris* SGB4557 that were present in three out of four responsive patients receiving donor 1 (Fig. 2B and 3A; Table S5). Of note, patient 1_25 showed limited evidence of transfer of donor-only SGBs despite being a responder (Fig. 3A). To examine this further, we reclassified SGBs as donor-only, patient-only, or both for that individual patient (i.e., excluding data from the other three patients). We found an improved level of transfer (Fig. S2A), driven by commensal SGBs initially classified as present in both donor and patient due to the detection in at least one of the three other patients receiving donor 1. Moreover, using this patient-specific assignment, we noted a lack of detection of many patient-only SGBs, including those such as *Sutterella wadsworthensis* (Fig. S2B), previously associated with a lack of response to FMT (17).

Next, we performed a similar analysis for patients receiving FMT from donor 2. Here, the number of donor-only SGBs was lower (Fig. 2B) due to the inclusion of a larger number of patients in the analysis (*n* = 11). Despite this, and in concordance with donor 2 microbiota engrafting better (Fig. 1D), we detected 15 donor-only SGBs in all four patients who went into remission (Fig. 2B), including *Faecalibacterium prausnitzii* SGB15332, *Clostridiaceae* SGB511, SGB25547, and SGB6749, *Dysosmobacter* SGB15053, and *Collinsella intestinalis* SGB14741, and 7 *Blautia* SGBs (Fig. 3A). A further 19 donor-only SGBs were present in at least three of the four patients (Fig. 2B). The majority of the 34 SGBs detected in at least three responders showed robust transfer in the seven non-responders as well (Fig. 3A), except for three: *Clostridium* SGB6179 (100% vs 42.8%), GGB3627 SGB4906 (100% vs 57.1%), and *Butyricimonas* SGB15260 (75% vs 42.8%). Analysis of donor-only SGBs common across both donors that were detected in at least one patient from each donor found *Clostridium* SGB6179 to have been transferred to all eight responders (Fig. 3B). Coupled with low transfer in non-responders, this highlighted the potential clinical relevance of this taxon in the context of disease remission in UC. To investigate the taxon further, we downloaded 115 available *Clostridium* genomes from Genome Taxonomy Database and performed an average nucleotide identity search using fastANI version 1.33 (38, 39). We found the taxon to have 99.3% identity to *Clostridium* sp900540255, with the closest defined species being *Clostridium saudiense* at 85.5%.

We also explored patient-only SGBs that had differential prevalence among responders and non-responders. Several SGBs showed increased prevalence in non-responders, including *Eisenbergiella massiliensis* SGB4987 (100% vs 50%) and *Clostridium spiroforme* SGB6747 (57.1% vs 0%) (Fig. S3). In contrast, *Monoglobus pectinilyticus* SGB4166 was present in 100% of responders compared to 42.8% of non-responders.

### Long-term low-dose FMT therapy appears to be an effective maintenance strategy

**Fig 4:**
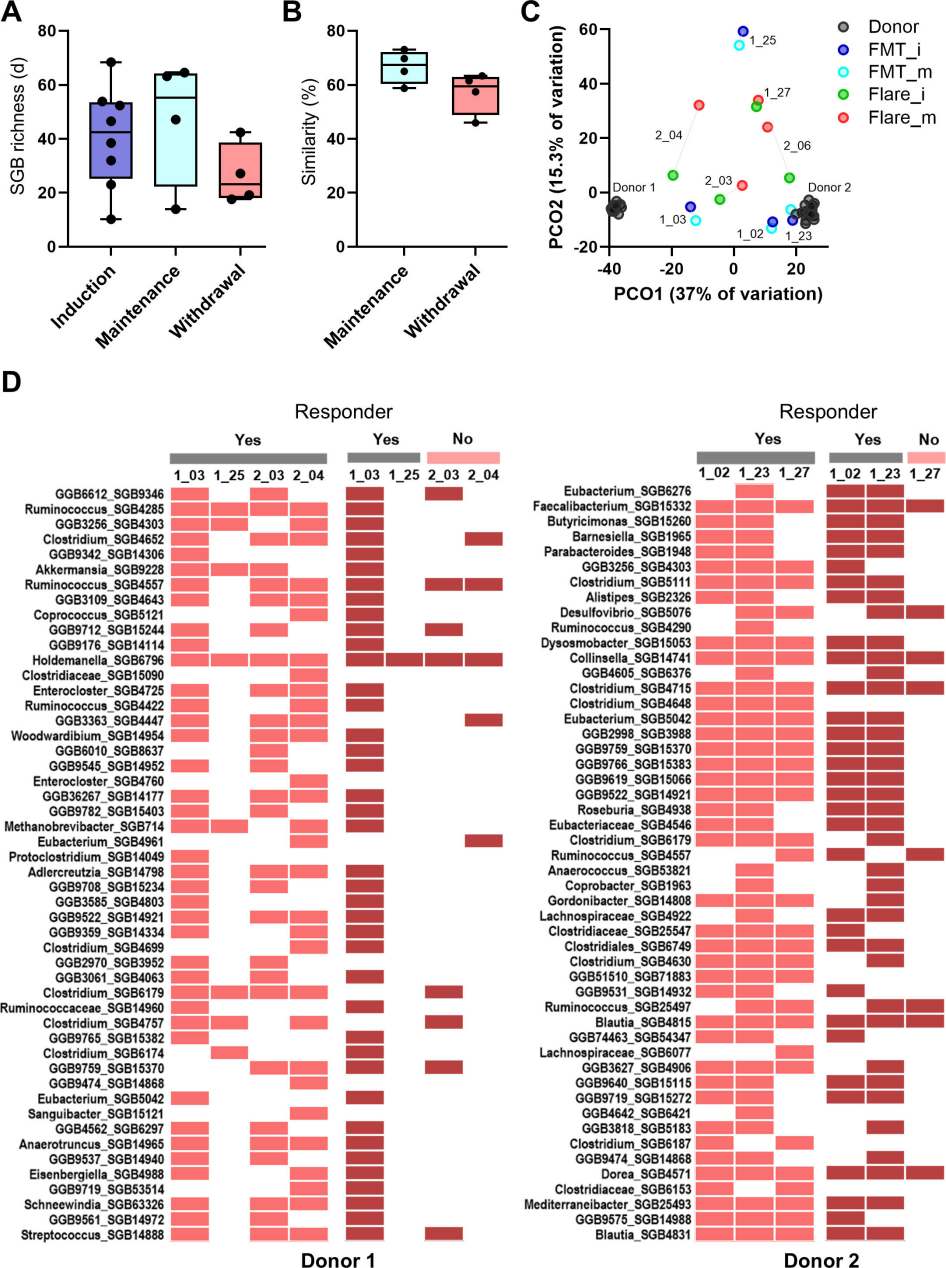
Taxonomic diversity following low-dose long-term maintenance therapy with oral lyophilized FMT. (**A**) Change in species genome-bin richness in patients on low-dose FMT vs those who withdrew from therapy compared to their SGB richness at the end of FMT induction therapy. Data distribution was tested with the Shapiro-Wilk test, and differences among groups were tested with one-way ANOVA with Tukey’s multiple comparison test (*F*_2,13_=1.314, *P* = 0.3021). (**B**) Pairwise similarities between each patient’s last sample within the induction arm and a sample from the maintenance/withdrawal arm. Similarity percentage was calculated using the following equation: 100 x (1 − Bray-Curtis dissimilarity). Differences were tested using an unpaired *t*-test (*t* = 1.922, *P* = 0.1030, and df = 6) after confirmation of data distribution with a Shapiro-Wilk test. (**C**) Changes in patient SGB composition following low-dose FMT or withdrawal. Ordination plot is a principal coordinate analysis on Bray-Curtis dissimilarities calculated from square-root transformed SGB relative abundances. Patients receiving low-dose FMT at induction (FMT_i) and end of maintenance (FMT_m); patients withdrawing from therapy and flaring at induction (Flare_i) and withdrawal (Flare_m). Statistical analysis of similarity to donors is reported in Fig. S4; Table S6. (**D**) Heatmap of detection of top 50 donor-only SGBs in patients following FMT as ranked by mean relative abundance at induction therapy. Light red, present at induction time point; dark red, present at maintenance or withdrawal time point; Yes, responder; No, non-responder; PCO1, principal coordinate axis 1; and PCO2, principal coordinate axis 2.

This clinical trial included a maintenance arm that randomized a subset of responsive patients to either low-dose FMT or withdrawal (Fig. 1A). Patients maintained on FMT were in clinical, endoscopic, and histologic remission at week 56, while patients withdrawn all flared within 6 months (21). While patients in the withdrawal group had lower SGB richness and those maintained on FMT had higher SGB richness when compared to patients at the end of induction therapy, the differences were not statistically significant (all pairwise *P* > 0.29; Fig. 4A). Moreover, patients maintained on FMT had a higher similarity to their week 8 samples than those withdrawn from therapy (*P* = 0.1; Fig. 4B; Fig. S4A). This is despite samples from those maintained on FMT on average being collected further apart from those withdrawn from FMT (35.0 ± 4.7 vs 27.0 ± 3.0 weeks). This was confirmed visually on principal coordinate analysis, where patients maintained on FMT remained close or moved toward the donors, whereas those who withdrew shifted away (Fig. 4C; Fig. S4B; Table S6). SGB-level features also supported this observation, with patients who received FMT maintaining many SGBs transferred during induction treatment, while those who withdrew and flared lost many of these SGBs irrespective of the donor received (Fig. 4D; Fig. S4C). Notably, the ordination plot supported our previous observation of low donor SGB transfer in patient 1_25 (the most distant induction sample from donors; Fig. 4C), but even in this patient with what appears to be a resistant microbiota, low-dose long-term FMT led to a small shift toward the donor (Fig. 4C) and improved donor-only SGB transfer (Fig. S2A).

### Specific functional pathways are associated with the response to FMT in patients with UC

**Fig 5:**
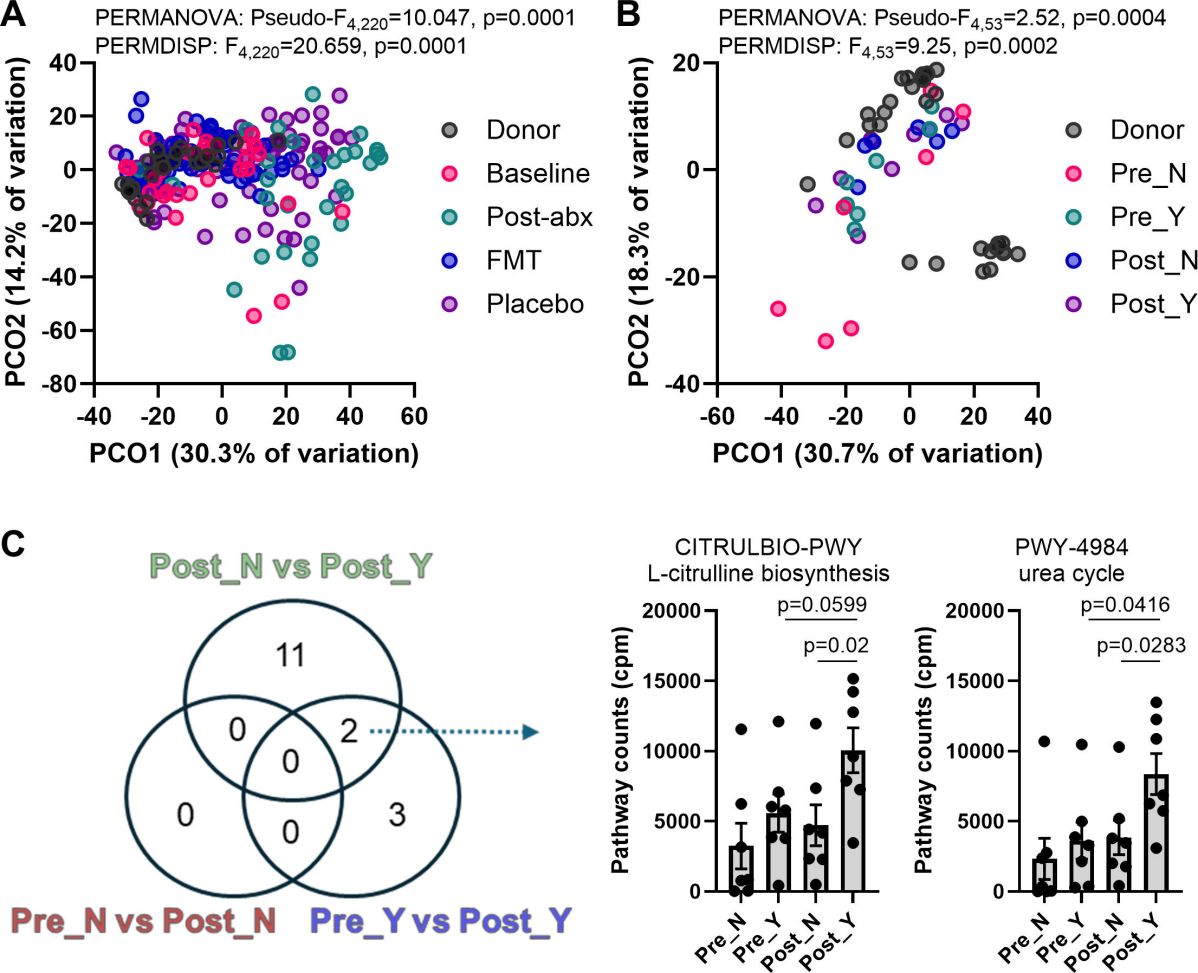
Functional changes in the gut microbiome of patients with UC receiving oral lyophilized FMT. (**A**) Changes in microbial functional composition following antibiotics, donor FMT, and placebo. Ordination plot is a principal coordinate analysis on Aitchison distances, with inter-group differences tested using PERMANOVA and PERMDISP. Pairwise comparisons are included in Table S8. (**B**) Changes in microbial functional composition in responders and non-responders before and after FMT. Ordination plot is a principal coordinate analysis on Aitchison distances, with inter-group differences tested using PERMANOVA and PERMDISP. Pairwise comparisons are included in Table S9. (**C**) Venn diagram of pathways identified to have significantly different relative abundances across three independent comparisons. Longitudinal analyses (Pre_N vs Post_N and Pre_Y vs Post_Y) were performed using Wilcoxon matched-pairs signed rank tests, while the cross-sectional analysis was performed using Mann-Whitney tests. Counts per million (cpm) of two microbial MetaCyc pathways identified to be associated with response were included. Validation statistical tests were performed on these two individual pathways in the form of a repeated-measures two-way ANOVA. Only multiple comparisons with *P* < 0.1 using Fisher’s LSD were reported. Post-abx, post-antibiotics; PCO1, principal coordinate axis 1; PCO2, principal coordinate axis 2; Pre_N, non-responders prior to FMT; Pre_Y, responders prior to FMT; Post_N, non-responders following FMT; and Post_Y, responders following FMT.

We next examined if antibiotic pre-treatment and oral lyophilized FMT influenced bacterial functional profiles in patients with UC (Fig. 5A; Table S7). Antibiotics led to a significant shift in the functional profile of the patients’ microbial communities (*t* = 3.27, *P* = 0.0001), with both FMT (*t* = 4.75, *P* = 0.0001) and placebo (*t* = 3.05, *P* = 0.0001) having significant effects post-antibiotics as well. While the latter likely resulted from incomplete recovery of the patients’ microbiotas, FMT shifted functional profiles to a composition between donors and patient baselines (Fig. 5A; Table S8). Stratification based on clinical response did not identify any differences in global functional composition between responders and non-responders (Fig. 5B; Table S9). However, further analysis of individual pathways identified two pathways that had altered relative abundances longitudinally in responders (i.e., baseline/Pre_Y vs post-FMT/Post_Y) and cross-sectionally between responders and non-responders following FMT (i.e., Post_N vs Post_Y) but not longitudinally in non-responders (i.e., Pre_N vs Post_N) (Fig. 5C). Longitudinal analyses were performed using Wilcoxon matched-pairs signed rank tests, while the cross-sectional analysis was performed using Mann-Whitney tests. The two pathways identified were the associated L-citrulline biosynthesis and the urea cycle (Fig. 5C), with the main classified bacterial contributors in our data being *Alistipes finegoldii* (both pathways) and *Alistipes onderdonkii* (L-citrulline biosynthesis).

### FMT depletes the resistome in patients with UC following antibiotics

**Fig 6:**
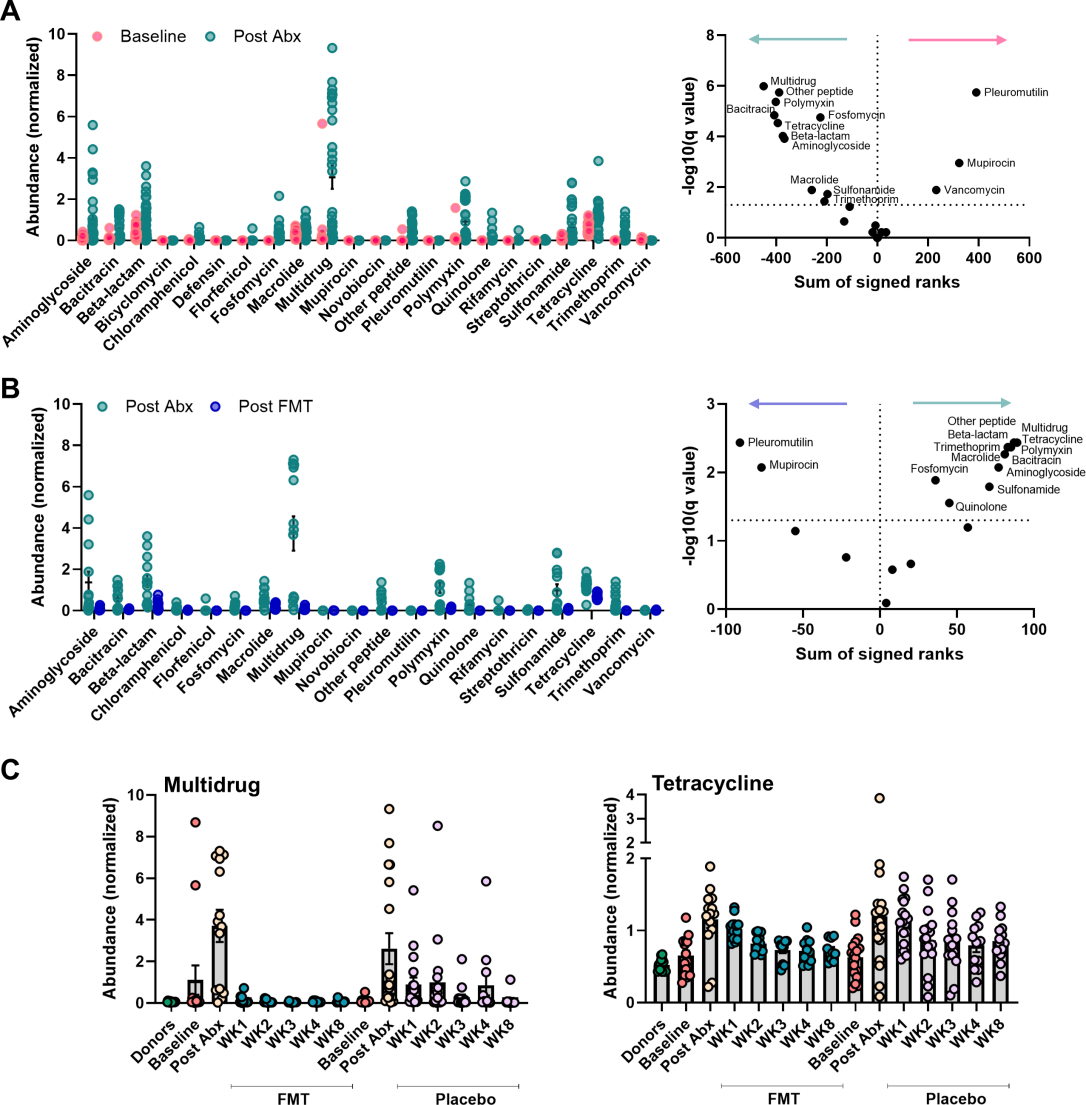
Changes to the resistome in patients receiving antibiotics and FMT. (**A**) Normalized abundances of antibiotic resistance types before and after antibiotics. Volcano plot of corrected *P*-values with direction of change was included (red, higher in baseline; green, higher post-antibiotics). (**B**) Normalized abundances of antibiotic resistance types before and after FMT. Volcano plot of corrected *P*-values with direction of change was included (green, higher post-antibiotics; blue, higher post-FMT). Differences for both comparisons were tested using the Wilcoxon matched-pairs signed rank test, and *P*-values were corrected for false discovery rate using the Benjamini and Hochberg method. The volcano plots show the corrected *P*-values on the *y*-axis and the sum of signed ranks on the *x*-axis for each antibiotic resistance type. Abundances are normalized against an estimated cell number by mapping against a database containing essential single-copy marker genes. (**C**) Normalized abundances of antibiotic resistance genes contributing to multidrug resistance and tetracycline resistance across treatment and time.

We analyzed the resistomes within the patients’ microbial communities in a longitudinal fashion to determine what changes occur following antibiotic treatment and FMT (Table S10). The 2-week triple-regimen antibiotic therapy (amoxicillin, metronidazole, and doxycycline) increased the normalized abundance of resistance genes for most antibiotics (Fig. 6A), the highest being multidrug resistance. Intriguingly, low levels of vancomycin resistance in patients at baseline decreased even further with antibiotic therapy (Fig. 6A), suggesting the targeting of a vancomycin-resistant microorganism in some patients. In contrast, FMT from healthy donors depleted the resistome of patients from resistance genes of most antibiotics, on many occasions to levels lower than baseline (Fig. 6B). Qualitatively, we also observed that multidrug resistance remained high in patients on placebo for longer, and higher dispersion was seen in patients on placebo for the common resistance type, tetracycline (Fig. 6C).

### Placebo responders may have benefited from antibiotic treatment

In this clinical trial, 3 of 20 patients responded to a placebo (15%), achieving the primary endpoint. We analyzed their microbiome profiles relative to non-responders on placebo to determine if this may reveal patient bacterial signatures associated with active disease, noting the limited power of this analysis due to low numbers. While no differences between the responders and non-responders were found for alpha and beta diversity (data not shown), three SGB level features revealed a unique pattern of presence in all responders at baseline and eradication with antibiotics (i.e., did not return on placebo; Fig. S5). Of the three features, *Faecalibacillus intestinalis* and *Ruminococcus lactaris* were not of particular interest, as the former was eradicated from eight of nine non-responders it was present in, while the latter was only present in three non-responders (eradicated from one) (Fig. S5). In contrast, *Ruminococcus torques* was present in 11 non-responders (total *n* = 15) and only eradicated in 3 (Fig. S5).

### Analysis of metagenome-assembled genomes validates transfer of donor taxa and functions to patients

**Fig 7:**
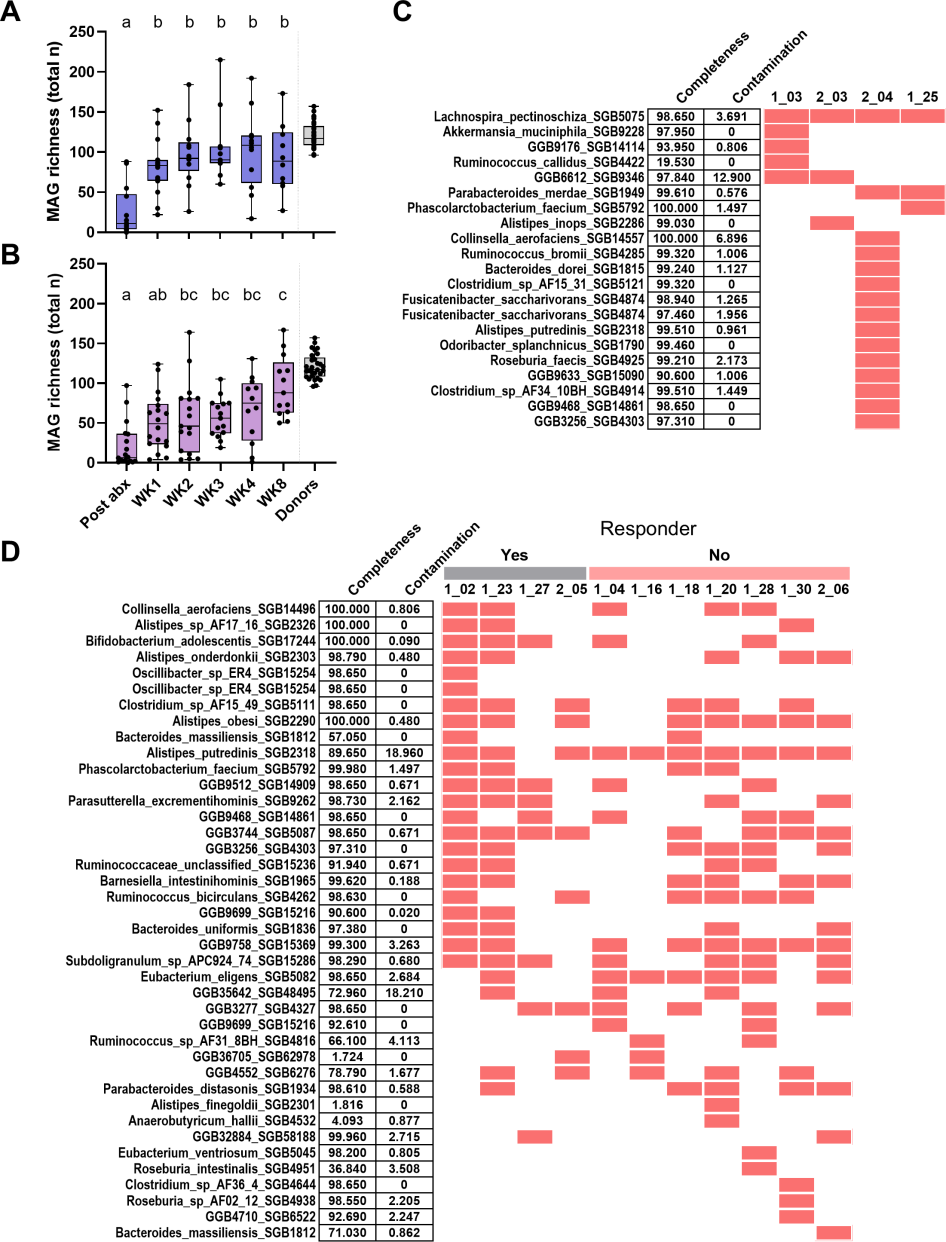
Oral lyophilized fecal microbiota transplantation alters the microbiome of patients with ulcerative colitis at the metagenome-assembled genome level. Changes in MAG richness (count, total number of MAGs) following (**A**) FMT or (**B**) placebo in patients with ulcerative colitis treated with antibiotics. Differences were tested using two-way ANOVA with a Tukey’s multiple comparisons test, combining data from both treatments (FMT and placebo). Only within-group statistics are presented as a compact letter display (different letters, significantly different; same letters, not significantly different), with the remaining statistics included in Table S13. Donors were excluded from statistical tests as they represent longitudinal samples from two individuals. (**C**) Heatmap of detection of MAGs in patients following FMT from donor 1. All MAGs for taxa were near complete except for *Ruminococcus callidus* SGB4422, GGB6612 SGB9346, and *Collinsella aerofaciens*. (**D**) Heatmap of detection of MAGs in patients following FMT from donor 2. All MAGs for taxa were high quality except for *Bacteroides massiliensis* SGB1812, *Alistipes putredinis* SGB2318, GGB35642 SGB48495, *Ruminococcus* SGB4816, GGB36705 SGB62978, *Alistipes finegoldii* SGB2301, *Anaerobutyricum hallii* SGB4532, and *Roseburia intestinalis* SGB4951. Red, present; Yes, achieved primary endpoint (i.e., responder); No, non-responder. Numbers above columns refer to de-identified patient codes. MAGs were classified using PhyloPhlAn3 version 3.0.60.

We generated and classified MAGs from each individual sample from donors and patients and then merged them into a presence/absence table across all samples using ANI thresholds of >99.99%, as previously suggested (40, 41) and at >97% (Tables S11 and S12). The 99.99% threshold generated 18,517 bins, of which 9,003 bins were of high and moderate quality (>50% complete and <10% contamination). The 97% cutoff generated 5,344 bins, of which 1,132 bins were high or moderate quality. We observed that MAG richness followed a similar pattern to that observed for SGB richness (Fig. 7A; Fig. S6A). The results were consistent when MAGs were de-replicated at 97% ANI, as well as when the analysis was restricted to high and moderate quality MAGs for either cutoff (Fig. S7A through C). We selected donor-only, patient-only, and mixed MAGs using a similar strategy to the above and tracked the presence or absence of donor-only MAGs in the microbiome of patients during FMT or placebo (all bins at 99.99% cutoff). Analysis of specific donor-only MAGs with evidence of transfer into patients’ microbiomes revealed a taxonomically diverse list of MAGs to be transferred, including several classified to *Clostridium* and *Alistipes* spp. (Fig. 7B and C), in line with the SGB-level analysis. A unique finding using the MAG-level analysis was the transfer of a MAG (98.65% complete) classified to *Lachnospira pectinoschiza* in all patients receiving donor 1 (Fig. 7B). We next performed a functional enrichment analysis using clusterProfiler version 4.16.0 (42). Overrepresented KEGG modules (adjusted *P* < 0.05) within the MAGs that transferred into patients (analyzed per patient) were identified using the predicted KEGG IDs, and only modules absent in at least one patient were included (Fig. S6B). Modules that were enriched in a higher number of the eight responsive patients when compared to the seven non-responders included fatty acid biosynthesis initiation (M00082; 87.5% vs 28.6%), phenylalanine biosynthesis (M00024; 50% vs 0%), and phylloquinone biosynthesis (M00932; 25% vs 0%). In contrast, vancomycin resistance, D-Ala-D-Lac type, was enriched in a higher number of non-responders (M00651; 25% vs 71.4%). In addition to the above, riboflavin biosynthesis (M00125; 100% vs 57.1%), ornithine biosynthesis (M00028; 75% vs 28.6%), and methylaspartate cycle (M00740; 75% vs 42.8%) showed enrichment in a higher number of responders (*n* = 4) than non-responders (*n* = 7) when the analysis was restricted to patients receiving donor 2.

## DISCUSSION

There is a limited amount of work examining the effects of oral lyophilized FMT on the microbiome of patients with UC, and less so studies examining dynamics at the SGB and MAG levels, as well as functional changes using this form of FMT. Here, we analyzed shotgun metagenomic sequencing data of fecal microbiomes of patients and donors recruited to the LOTUS study, a randomized, double-blind, placebo-controlled clinical trial of single-donor, oral, lyophilized FMT. We found that oral lyophilized FMT can significantly alter the diversity and composition of the patient microbiome at the SGB and MAG levels, with changes seen by the first week of therapy. We did not find an association between SGB or MAG richness and clinical response in this trial, but transfer of a specific *Clostridium* SGB (SGB6179) to patients, as well as enrichment of L-citrulline biosynthesis contributed by *Alistipes* spp., was features of responders. We observed that low-dose long-term FMT within the maintenance arm was effective at retaining donor SGBs in patients. We also found that the response to the placebo may have been a consequence of prior eradication of *R. torques* SGB4563 with antibiotics, but this requires further investigation. Moreover, we showed that FMT can deplete the resistome of patients treated with antibiotics, particularly impacting important antibiotic resistance types such as multidrug resistance.

While advancements in microbial-based therapies have been made, the options remain limited to a few candidates currently undergoing testing (43). However, outside the research environment, the key aspects to achieve to ensure general applicability of these therapeutics would be stability during storage and delivery, as well as ease of use. To this end, lyophilization and encapsulation of microbial strains would assist in overcoming these issues. We show that following a triple regimen of antibiotics, lyophilized FMT in capsules delivered orally is effective at modifying the microbiome of patients with UC at the SGB level, not only at induction therapy but also during low-dose maintenance therapy. This is in line with our findings in *Clostridioides difficile* infection, where this form of FMT could cure patients by engrafting donor bacteria (44). It is also in line with previous studies that used oral FMT as an induction therapy (45) or a long-term maintenance therapy (46) for patients with UC, both showing donor bacteria engraftment in patients. However, both of the above studies utilized frozen FMT, with one reporting home storage concerns (46), which could be overcome with the use of lyophilized material.

We find that one of the donor’s bacteria more readily influenced patients’ microbiotas during induction therapy, being more effective at shifting patient microbiomes to the donor profile, as well as having a larger number of SGBs and MAGs transferring into patients. Notably, this donor was the least effective donor in the clinical trial, contributing seven non-responders (21). This demonstrated that despite rigorous selection of healthy donors, transfer of donor taxa is only one factor influencing outcome in the context of UC. Another factor is the identity and function of what is transferred, with many donor- and patient-specific changes seen in responders to therapy. Despite this, we did identify commonalities across both donors and responders, including the transfer of a *Clostridium* SGB (SGB6179) in all responders from both donors. Little is known about *Clostridium* SGB6179, but it appears to be a prevalent taxon within the gut microbiome of healthy individuals (25) and a stable feature of the gut microbiome of patients with melanoma who respond to anti-PD-1 therapy (47). We also found L-citrulline biosynthesis contributed by *Alistipes* spp. to be enriched in responders. Plasma levels of L-citrulline have been associated with intestinal function and lower gastrointestinal disease severity on meta-analysis (48). Moreover, L-citrulline supplementation was shown to have protective effects in two animal models of chemically induced colitis (49, 50), decreasing immune cell infiltration and improving barrier integrity. The bacterial species contributing to this pathway in our study, *A. finegoldii* and *A. onderdonkii,* belong to a genus reported to have protective effects in colitis (51). In addition, the MAG-level analysis for patients receiving donor 2 revealed that riboflavin biosynthesis was a functional feature that distinguished donor MAGs transferring to responders when compared to those transferring to non-responders. In addition to its role as a vitamin, riboflavin is utilized by various bacteria for growth, including the abundant commensal bacterium *Faecalibacterium prausnitzii*, a known butyrate producer and inulin fermenter (52).

While the number of patients in the maintenance arm of the study was low, leading to non-significant differences, we did find that long-term low-dose FMT maintained donor SGB presence, while withdrawal from therapy led to a loss of donor SGBs. This was supported by observations that samples from patients withdrawn from therapy became less similar to their own samples at induction and also shifted away from the donors. Collectively, this supports a role for donor SGB transfer, even if short term in nature, in inducing remission. It also highlights the importance of maintenance therapy for patients with UC to maintain clinical response and avoid disease flares. This is consistent with our findings from a 5-year follow-up study of a previous clinical trial, which showed, on average, patients with UC previously treated with FMT flared around 6 months after the conclusion of therapy (53).

Not many studies have examined the resistome (antibiotic resistance genes within the microbiome) in patients with UC, and those that have report conflicting findings (54, 55). Given that antibiotic pre-treatment of patients prior to FMT is suggested to make their microbiome more conducive to donor taxa engraftment, we examined how the resistome of patients behaves following antibiotics and FMT. We showed that antibiotics resulted in an increase in the relative abundance of antibiotic resistance genes within the microbiome profiles. While this is to be expected considering antibiotic treatment in this clinical trial consisted of a broad triple regimen for 2 weeks, the level of increase of notable antibiotic resistance types like multidrug resistance was striking. However, we also found that FMT was sufficient at depleting the resistome post-antibiotics, not only to levels at baseline in UC but even lower. This may be the result of patients with UC having increased exposure to antibiotics over their lifetime when compared to healthy individuals, and antibiotics being a risk factor for disease onset (56). These findings support previously reported observations that FMT holds promise for reducing the colonization of patients by antibiotic-resistant organisms, as well as for the treatment of drug-resistant gastrointestinal infections (57–60). However, these findings should be interpreted with caution, as they may simply reflect taxonomic shifts toward increased or decreased relative abundance of bacteria that more frequently carry antibiotic resistance genes.

Our analysis of responders on placebo identified the eradication of the mucin degrader *R. torques* by antibiotics as a potential discriminating feature from non-responders on placebo. While this bacterium has been previously reported to be depleted in patients with inflammatory bowel diseases (61), other studies have shown it to be increased in prevalence and abundance across the two major subtypes of inflammatory bowel diseases (62–65). Despite possessing several beneficial functions, one potential mechanism for its contribution to gastrointestinal pathologies is its ability to degrade mucin effectively (66), which, in susceptible individuals with an already altered gut mucus layer, such as in patients with UC, may impact barrier integrity. This context-dependent detrimental impact has been previously shown for another gut mucus degrader, *Akkermansia muciniphila* (67). However, this analysis should be taken with caution as only three patients responded while on placebo.

The study has several limitations. The clinical trial was terminated early due to the COVID-19 pandemic, leading to a lower number of patients recruited than initially planned. As a result, the extended longitudinal analysis has a limited number of patients, and the outcomes should be taken with caution. There are documented issues with computational assignment of bacterial SGBs or MAGs without validation by culture, and these may have an impact on the study. We employed heavily utilized software in the field with the latest database available at the time to ensure reproducibility. Moreover, as the patients receiving induction FMT and then maintenance FMT were not followed up at a time point without FMT, it is not possible to establish if donor SGBs in their microbiota are transient, have been transferred in the short term, or have permanently engrafted. However, data from patients who withdrew from FMT indicated that donor SGBs were present in follow-up samples, suggesting some level of colonization, which is supported by previous studies in *Clostridioides difficile* infection where donor bacteria were detected 6 months after treatment with oral lyophilized FMT (44), and in UC where donor strains were detected 5 years after FMT (68).

Overall, we find that single-donor oral lyophilized FMT given after a course of antibiotics can substantially modify the microbiome and resistome in patients with UC, with *Clostridium* SGB6179 and L-citrulline biosynthesis being two pan-donor and pan-patient signatures associated with response to FMT.

## Data Availability

The shotgun metagenomic sequencing data from the LOTUS donors used in this study are available from ENA under the accession number PRJEB50699 (https://www.ebi.ac.uk/ena/browser/view/PRJEB50699). Shotgun metagenomic sequencing data from the LOTUS patients have been previously submitted to ENA under the accession number PRJEB58035 (https://www.ebi.ac.uk/ena/browser/view/PRJEB58035). Additional metadata are available in Table S1. Relevant code has been deposited into Zenodo and can be accessed with the DOI: 10.5281/zenodo.16635322. All other software versions and operating procedures have been cited in the Methods. STORMS checklist: DOI: 10.5281/zenodo.14997110.

## SUPPLEMENTAL MATERIAL

Supplemental Figures msystems.00991-25-s0001.docx Figures S1 to S7.

Supplemental Tables msystems.00991-25-s0002.xlsx Tables S1 to S13.

ASM does not own the copyrights to Supplemental Material that may be linked to, or accessed through, an article. The authors have granted ASM a non-exclusive, world-wide license to publish the Supplemental Material files. Please contact the corresponding author directly for reuse.
