# Supplementary material for: Bacterial taxonomic and functional changes following oral lyophilized donor fecal microbiota transplantation in patients with ulcerative colitis
Source: mSystems. 2025 Sep 15;10(10):e00991-25. doi: 10.1128/msystems.00991-25 (PMC12542657; doi:10.1128/msystems.00991-25)

**Bacterial taxonomic and functional changes following oral lyophilized donor fecal microbiota transplantation in patients with ulcerative colitis**

Shreeya S Raich^1#^, Marwan E Majzoub^1#^, Craig Haifer^2,3^, Sudarshan Paramsothy^4,5^, Md Mushahidul Islam Shamim^1^, Thomas J Borody^6^, Rupert W Leong^4,5^, Nadeem O Kaakoush^1^*

^1^School of Biomedical Sciences, Faculty of Medicine and Health, UNSW Sydney, NSW 2052, Australia

^2^School of Clinical Medicine, Faculty of Medicine and Health, UNSW Sydney, NSW 2052, Australia

^3^Department of Gastroenterology, St Vincent’s Hospital, Sydney, NSW 2010, Australia

^4^Concord Clinical School, University of Sydney, Sydney, NSW 2006, Australia

^5^Department of Gastroenterology, Concord Repatriation General Hospital, Sydney, NSW 2139, Australia

^6^Centre for Digestive Diseases, Sydney, NSW 2046, Australia

^#^ These authors contributed equally

**Correspondence:**

Scientia A/Prof Nadeem O Kaakoush

School of Biomedical Sciences, Faculty of Medicine and Health

University of New South Wales, Sydney, NSW 2052, Australia

Tel: + 61 2 9032 9728; Email: [n.kaakoush@unsw.edu.au](mailto:n.kaakoush@unsw.edu.au)

**Running Title**

Microbiota transplantation in ulcerative colitis

**Supplementary Figure 1**

**Oral lyophilized fecal microbiota transplantation alters the microbiome of patients with ulcerative colitis.** **A:** Changes in species genome bin (SGB) richness following FMT or placebo in patients treated with antibiotics. Differences were tested using two-way ANOVA with a Tukey’s multiple comparisons test combining data from both treatments (FMT and placebo). Inter-group statistics are presented, with the remaining results included in Supplementary table 3. **B:** Changes in patient SGB composition following antibiotics, donor FMT and placebo. Ordination plot is a principal coordinate analysis on Aitchinson distances, with differences tested using PERMANOVA and PERMDISP. Baseline samples are stratified according to the treatment received (FMT: Baseline-F; placebo: Baseline-P). Baseline groups are not significantly different on pairwise PERMANOVA (t=0.97, p=0.5176). **C:** Mean of Aitchinson distances between final patient post FMT sample available and samples of donor they received. Data distribution was tested using the Shapiro-Wilk test (p=0.0038 for Donor 2) and inter-group distances were tested using a Mann-Whitney test. Post abx, post antibiotics; WK, week; PCO1, principal coordinate axis 1, PCO2, principal coordinate axis 2.


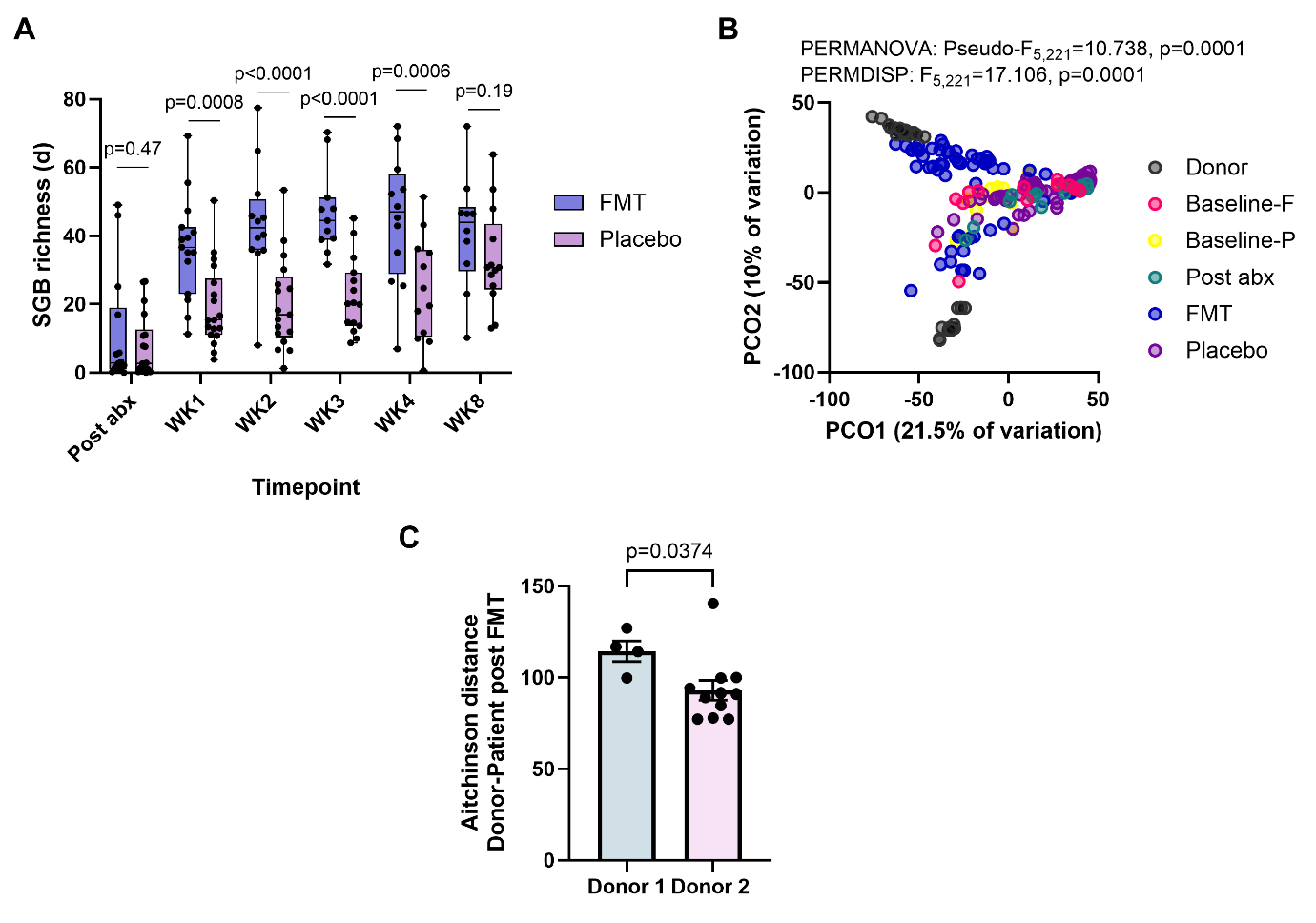


**Supplementary Figure 2**

**Presence of donor-only and patient-only species genome bins (SGBs) in patient 1_25. A:** Heatmap of detection of top 50 donor-only SGBs in patient 1_25 following induction and maintenance FMT as ranked by mean relative abundance. Donor-only SGBs were defined as those with no evidence of detection in this patient only. **B:** Heatmap of detection of patient-only SGBs in patient 1_25 at baseline, post antibiotics (post abx), following induction and maintenance FMT. Patient-only SGBs were defined as present at either baseline or post antibiotics but not in donor 1 and ranked by mean relative abundance. Red, present.

**
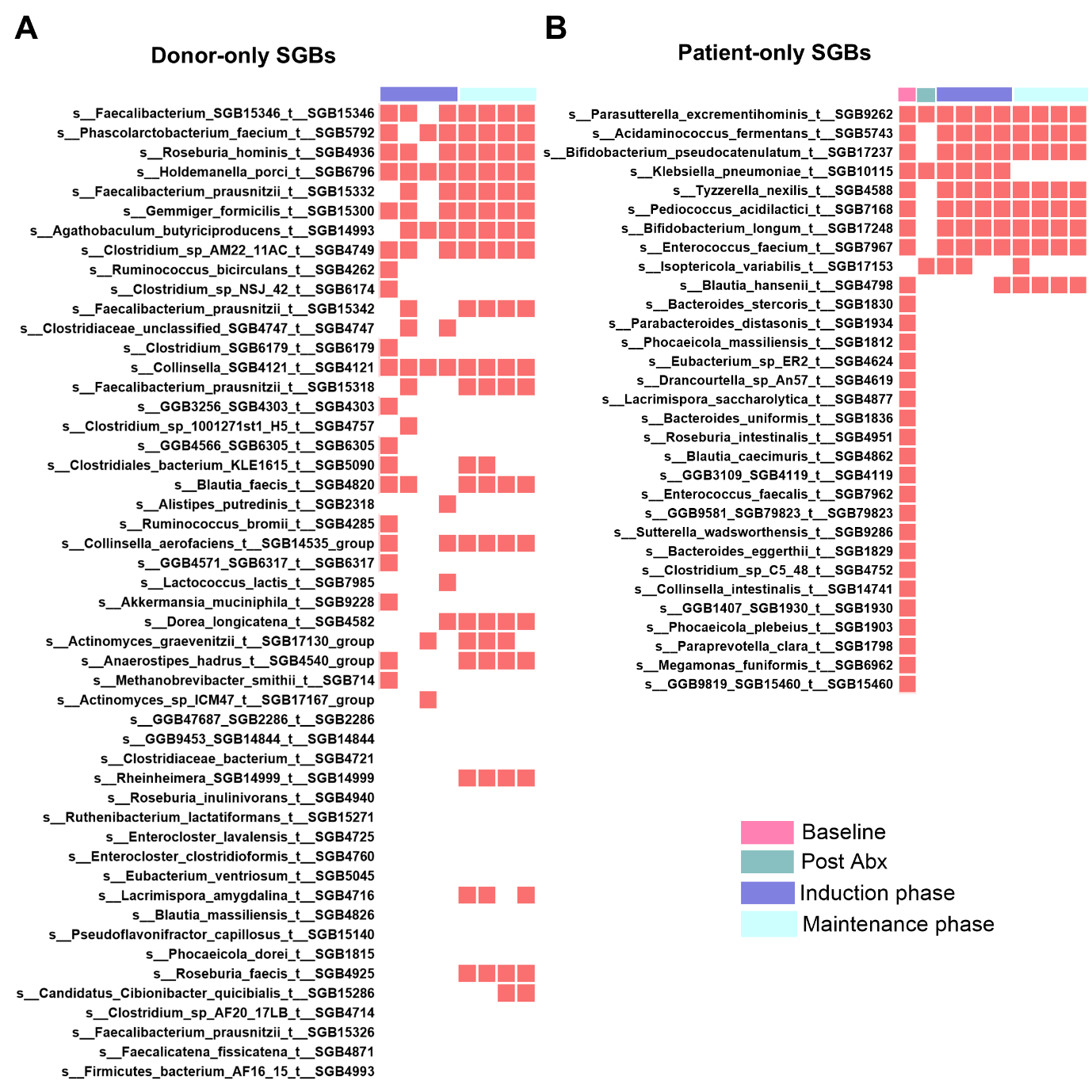
**

**Supplementary Figure 3**

**Patient-only species genome bins (SGBs) for patients receiving donor 2.** Patient-only SGBs were defined as present at either baseline or post antibiotics in patients but not in donor 2. Red, present; Yes, responder; No, non-responder.

**
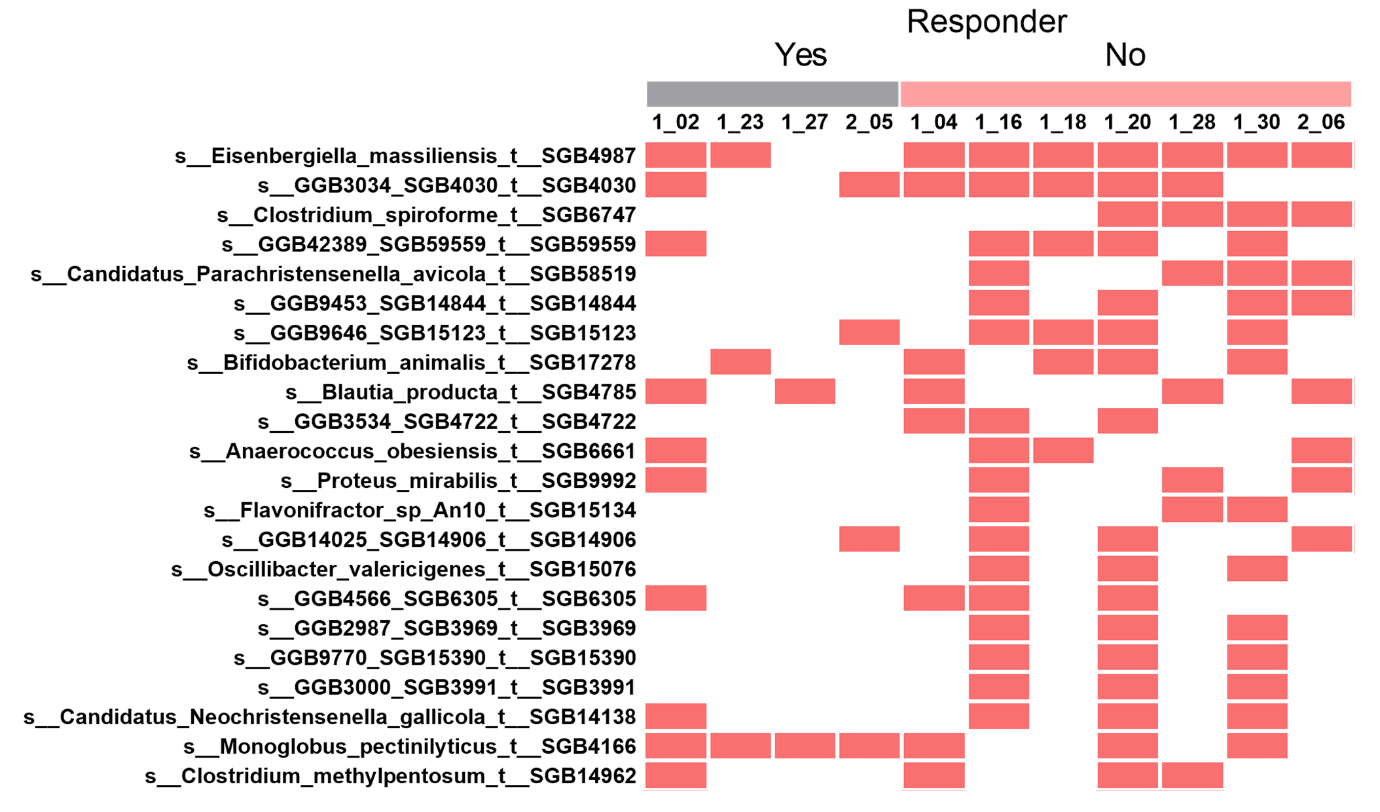
**

**Supplementary Figure 4**

**Microbiome changes following low-dose FMT or withdrawal. A:** Change in species genome bin (SGB) richness in patients on low dose FMT vs those that withdrew from therapy and flared across time (WK, weeks). **B:** Change in similarity to donors. Similarity was the mean of similarities to all samples from donor the patient received, and these were calculated as 100 x (1- Bray-Curtis dissimilarity). Bray-Curtis dissimilarity was calculated using square-root transformed relative abundances of SGBs. Test was a repeated-measures (RM) two-way ANOVA with the main test reported here and the multiple comparison tests reported in Supplementary table 6. Treatment: FMT (i.e., low dose FMT) vs Flare (i.e., withdrawal); Arm: Induction phase vs Maintenance phase; Treatment x Arm: interaction effect; subject: patient. **C:** Average number of events across treatment types. Gain refers to a patient gaining a SGB during the maintenance arm (FMT or Flare; Mann-Whitney test) whereas loss refers to a patient losing a SGB (unpaired t-test; t=8.427, df=5). Error is standard error of mean.


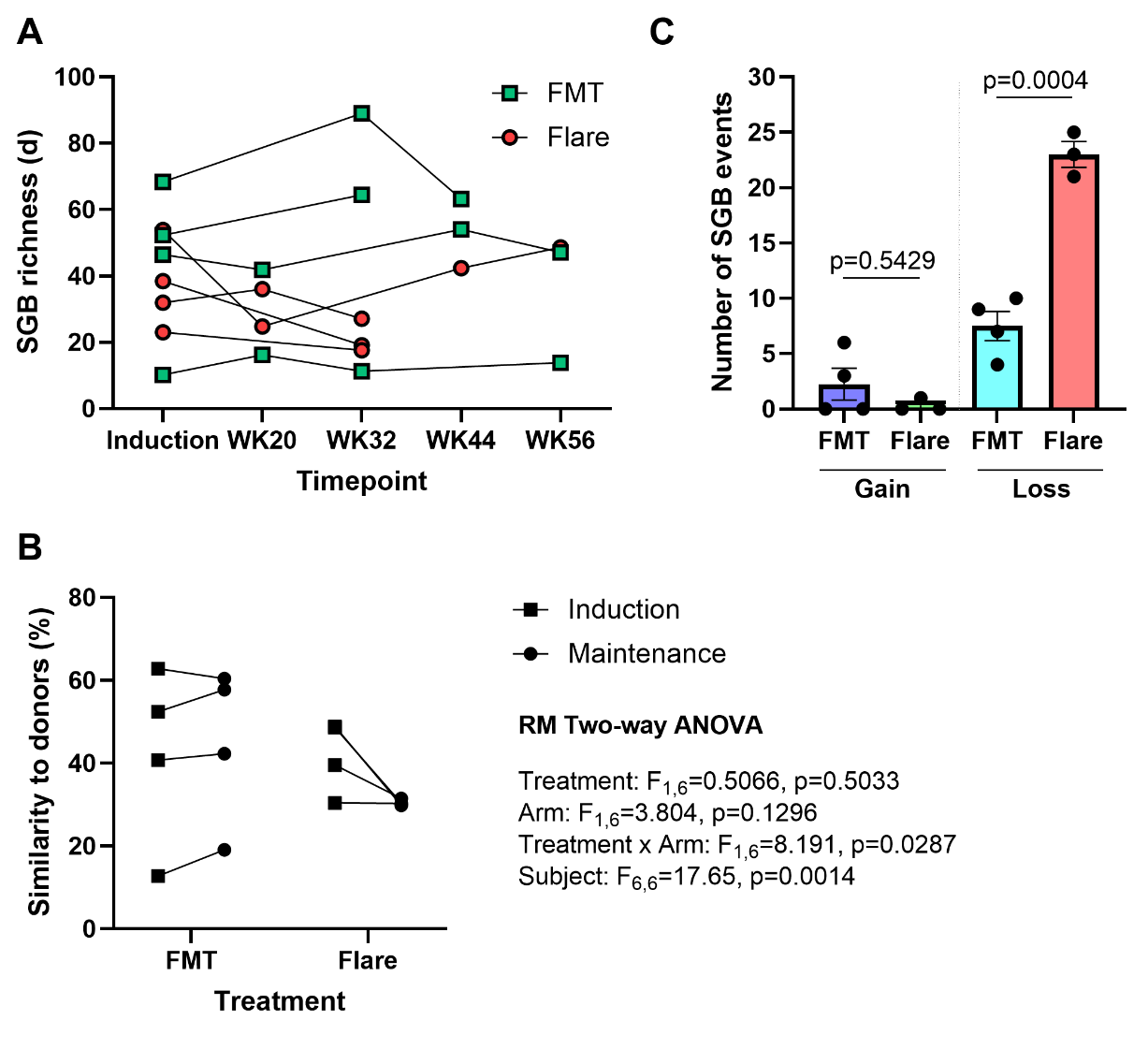


**Supplementary Figure 5**

**Patient-only species genome bins (SGBs) showing eradication post antibiotics in the three patients on placebo that responded.** Presence of these SGBs in non-responders on placebo was also included. Red, present; Post Abx, post antibiotics; Yes, responder; No, non-responder.


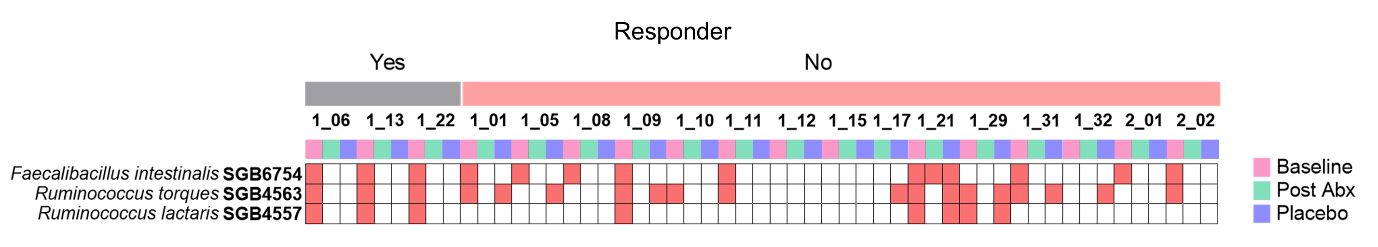


**Supplementary Figure 6**

**Oral lyophilized fecal microbiota transplantation alters the microbiome of patients with ulcerative colitis. A:** Changes in metagenome-assembled genome (MAG) richness following FMT or placebo in patients treated with antibiotics. Differences were tested using two-way ANOVA with a Tukey’s multiple comparisons test combining data from both treatments (FMT and placebo). Inter-group statistics are presented, with the remaining results included in Supplementary table 13. **B:** KEGG modules found to be significantly enriched (adjusted p<0.05) in predicted genes from donor-only MAGs that showed evidence of transfer to patients. Modules significantly enriched across all patients were not presented. Red, enriched. Numbers above columns refer to de-identified patient codes.


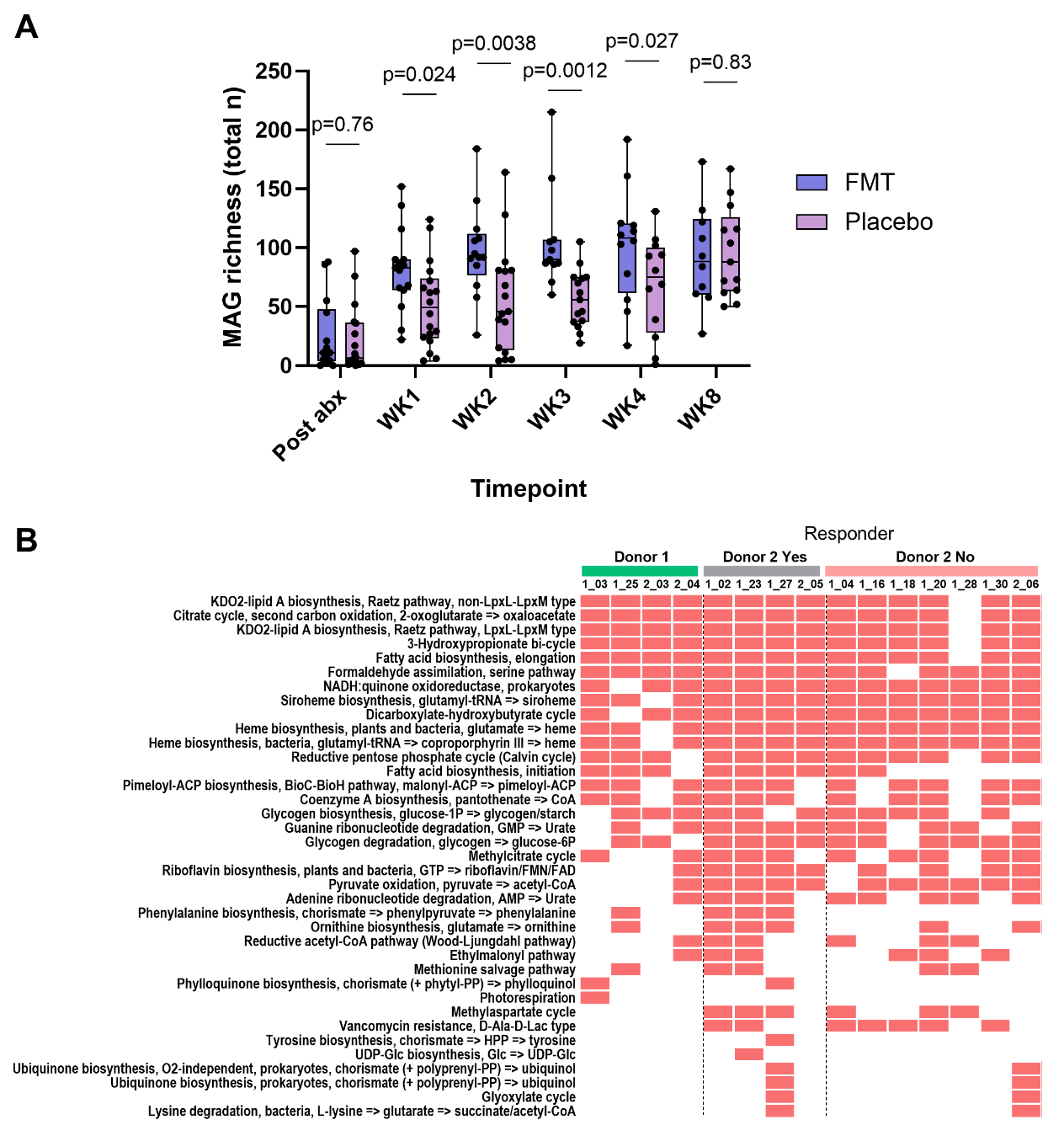


**Supplementary Figure 7**

**Oral lyophilized fecal microbiota transplantation alters metagenome-assembled genome (MAG) richness at various thresholds. A:** Changes in richness of high and moderate quality MAGs dereplicated at 99.99% average nucleotide identity (ANI) following FMT or placebo in patients treated with antibiotics. **B:** Changes in richness of MAGs dereplicated at 97% ANI following FMT or placebo in patients treated with antibiotics. **C:** Changes in richness of high and moderate quality MAGs dereplicated at 97% ANI following FMT or placebo in patients treated with antibiotics. Differences in all panels were tested using two-way ANOVA with a Tukey’s multiple comparisons test combining data from both treatments (FMT and placebo). Inter-group statistics are presented.


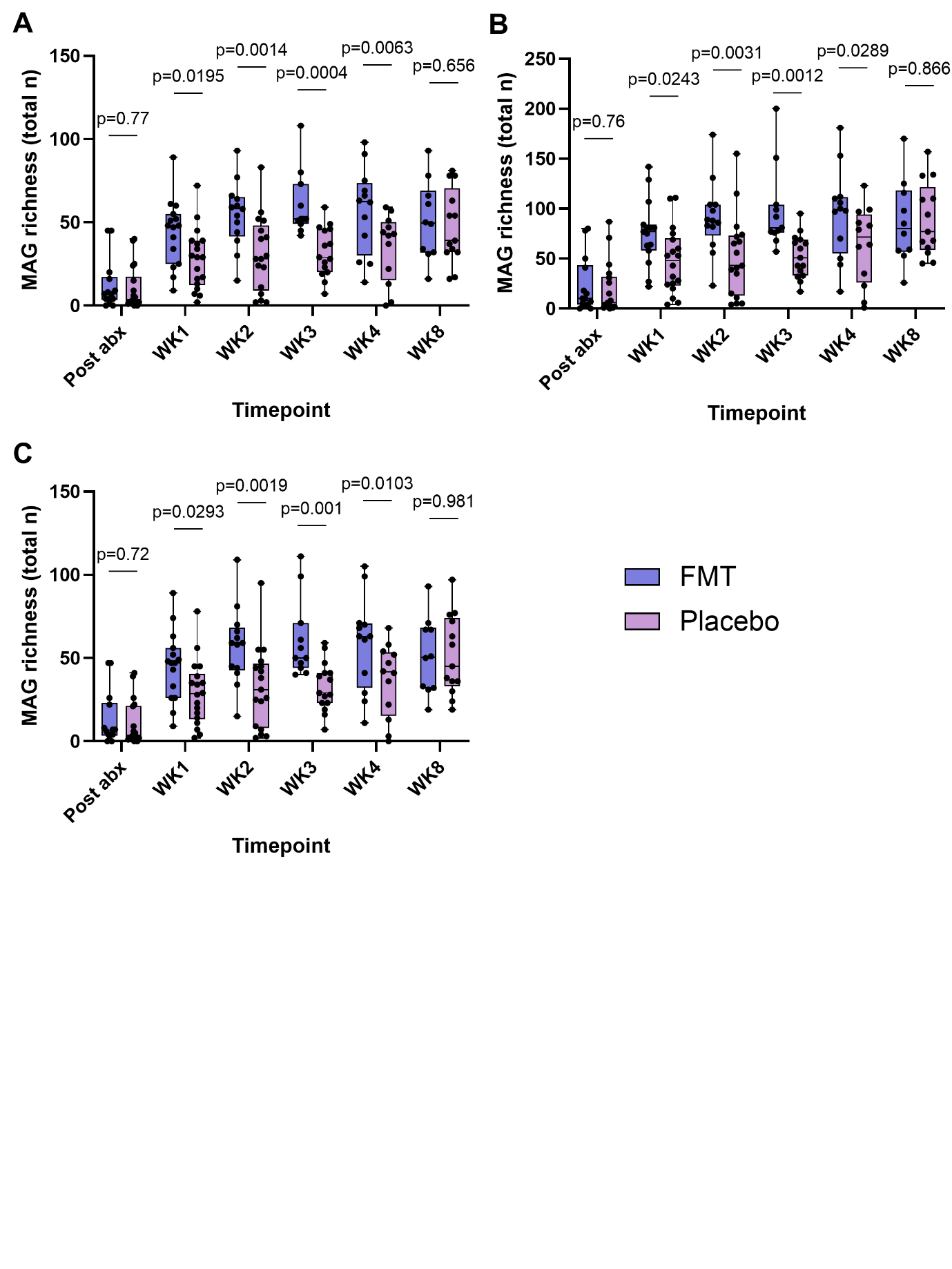

Supplement: Supplemental Figures — Figures S1 to S7. [file msystems.00991-25-s0001.docx]
